# Concomitant Detection of HER2 Protein and Gene Alterations by Immunohistochemistry (IHC) and Silver Enhanced In Situ Hybridization (SISH) Identifies HER2 Positive Breast Cancer with and without Gene Amplification

**DOI:** 10.1371/journal.pone.0105961

**Published:** 2014-08-25

**Authors:** Zsuzsanna Varga, Raymond R. Tubbs, Holger Moch

**Affiliations:** 1 Institute of Surgical Pathology, University Hospital Zurich, Zurich, Switzerland; 2 Section of Molecular Pathology, Cleveland Clinic, Cleveland, Ohio, United States of America; Istituto dei tumori Fondazione Pascale, Italy

## Abstract

**Introduction:**

HER2 status assessment became a mandatory test assay in breast cancer, giving prognostic and predictive information including eligibility for adjuvant anti-HER2 therapy. Precise and reliable assessment of HER2 status is therefore of utmost importance. In this study we analyzed breast cancer samples by a novel technology for concomitant detection of the HER2 protein and gene copy number.

**Methods:**

Tissue microarrays containing 589 invasive breast cancer samples were analyzed with a double immunohistochemistry (IHC) and silver labeled in situ hybridization (SISH) assay simultaneously detecting HER2 protein and gene copy number in the same tumor cells. This bright-field assay was analyzed using scores according to the modified ASCO guidelines and the results were correlated with patient prognosis.

**Results:**

Overall concordance rate between protein expression and the presence of gene amplification was 98%. Fifty-seven of 60 tumors (95%) with IHC score 3+, 6 of 10 tumors with IHC score 2+ (60%) and only 3 of 519 tumors (0.6%) with IHC score 0/1+ were amplified by SISH. Patients with gene amplification despite IHC score 0/1+ had a tendency for worse overall survival (p = 0.088, reaching nearly statistical significance) compared to IHC score 0/1+ without amplification. In contrast, there was no difference in overall survival in IHC score 3+/2+ tumors with and without gene amplification.

**Conclusions:**

The novel double IHC and SISH assay for HER2 is efficient in the identification of breast cancer with discordant HER2 protein and *HER2* gene status, especially for the prognostically relevant groups of HER2 protein negative tumors with HER2 amplification and HER2 protein positive tumors without HER2 amplification. Breast cancer without *HER2* amplification among IHC score 2+/3+ tumors (10% in our cohort) suggests that other mechanisms than gene amplification contribute to protein overexpression in these cells.

## Introduction

Determination of *HER2* status in breast cancer is important in the diagnosis of breast breast cancer by pathologists [1]–[6]. Accurate detection of the *HER2* protein and/or gene amplification is of enormous importance, as adjuvant or neoadjuvant therapy decisions, made on the assays, all rely on the scores that are given by these tests [4], [5], [7].

The ASCO guidelines approved several test assays for the HER2 status assessment including immunohistochemistry (IHC) and/or in situ hybridization (ISH) technics with different visualization methods as fluorescence (FISH), silver (SISH) or chromogenic (CISH) labeled probes [2], [5], [6]. These tests are conducted in a single way, either immunohistochemistry complemented with ISH technology or the ISH technology alone. In the literature, a good correlation could be found between standardized IHC and ISH technology, justifying the use of only one of these technologies in the routine diagnostic service. Furthermore, correlation of test results obtained by IHC and/or ISH tests, was proven to correlate with therapy response and with overall survival in several previous studies, confirming the prognostic and predictive value of these test assays [8]–[11]. A novel bright-field assay for concomitant detection of HER2 gene status and protein expression on one tissue section has been described by Tubbs et al. [12]. This assay allows the identification of tumor cells with discordant HER2 results, e.g. HER2 protein overexpression without evidence for *HER2* gene amplification.

In this study, we addressed the question whether such tumors with discordant results by IHC and SISH represent prognostically relevant subgroups. We are not aware of any previous studies, correlating results of this bright-field assay with patient prognosis [12], [13].

## Materials and Methods

### Patients' cohort

Tissue microarrays (TMAs) containing eligible 652 samples from breast cancer patients were selected for this study. Eligibility was decided upon availability of sufficient tumor tissues in the paraffin blocks. The TMAs were constructed previously as described [14]. Clinical data, at least five years follow-up and complete histopathological parameter including hormone receptor status (ER, PR), histological grading were available to all patients, tumor stage and the presence of local or distant recurrence were available for most of the patients.

The cohort was composed of 318 primary breast cancer (49%), 26 local recurrences (4%), 258 axillary lymph node metastases (39%) and 50 visceral metastases (8%). For the TMA analysis there were 589 of 652 interpretable samples available.

Histologically, there were 280 invasive ductal (88%), 35 invasive lobular carcinomas (11%) and 5 special types (1%). Grading was assessed by applying the modified Bloom and Richardson grading system after Ellis and Elston on the primary tumors and on most of the metastatic and recurring lesions [15], 89 cases were Grade 1 (13%), 318 cases were Grade 2 (49%) and 213 cases Grade 3 (33%), in 32 cases (5%) grading was not available in the metastastic lesions. The retrospective study on human tissue samples was approved by the Cantonal Ethical Committee of Zurich (KEK-2012-553). Informed consent was not necessary, as the ethical approval covered the ethical issues of the retrospective study and the samples were completely anonymized and de-identified prior to the study.

Details of clinico-pathological parameters are depicted in **Table 1**.

**Table 1:** Clinico-pathological characteristics of samples in the tissue micro arrays.

	n	%
Type of tissue samples (n = 652)		
Primary breast cancer	318	49%
Recurrent lesion	26	4%
Axillary lymph node metastasis	258	39%
Visceral metastasis	50	8%
Histological grade (all lesions)		
G1	89	13%
G2	318	49%
G3	213	33%
Missing	32	5%
pT stadium (all lesions)		
pT1	182	24%
pT2	301	40%
pT3	41	6%
pT4	101	13%
missing	128	17%
pN stadium (all lesions)		
pN0	39	6%
pN1	429	62%
missing	220	32%
ER/PR status (primary tumor)		
Positive	254	80%
Negative	64	20%
HER2 status (primary tumor)		
positive	28	9%
negative	290	91%
Histological type (primary tumor)		
Invasive ductal	280	88%
Invasive lobular	35	11%
Other type	3	1%

### Concomitant detection of HER2 protein expression and *HER2* gene copy number

For the simultaneous detection of the HER2 protein and HER2 gene copy number, we used a previously described protocol by Tubbs et al and Downs-Kelly et al. [12], [13]. The idea of this technology is a metallographic based procedure which comprises an enzymatic interaction between peroxidase or other enzymes and silver or other metal associated substrates. These interactions result in the deposition of the given metallic substrates at the corresponding protein site or gene locus within the cell. The best results were achieved by the combination of peroxidase and silver linked substrates [12], [13]. The procedure was carried out as follows: Unstained slides of 4 micrometer thickness were prepared from the paraffin block of the TMAs, one of them stained for routine hematoxylin & eosin to confirm the presence of sufficient amount of invasive tumor cells in the spots. One unstained slide underwent subsequent immunohistochemical treatment with the CB11 clone of the HER2 protein combined with metallic silver deposition of EnzMet GenePro completed with alkaline phosphate and fast red K substance for visualization according to the manufacture's recommendations (all reagents were purchased by Ventana Tucson, AZ, USA). Additionally, the Inform *HER2* dual DNA Probe Cocktail with the UltraView SISH Detection Kit (*HER2* locus) and UltraView Red ISH DIG Detection Kit (*CEP17* locus) were done on the same tissue micro arrays. Reactions mentioned above in both detection systems (except the hybridization step) were carried out on the automatic Benchmark system (Ventana, Tucson, AZ, USA). The hybridization step (containing the HER2 and CEP17 gene locus on chromosome 17) was carried out manually according to the instruction of the EnzMettrade mark, Nanoprobes (Yaphank, NY, USA) and on the automatic Benchmark ULTRA system (Ventana, Tucson, AZ, USA). The HER2 protein was visualized as a purple-red (EnzMet) or DAB brown (Inform) membranous stain, the gene loci (*HER2*) as confluent black aggregates or singular black spots, the *CEP17* region with red spots or aggregates [12], [13].

### Scoring of HER2 protein and *HER2* gene copy number

The simultaneous detection of the HER2 protein and HER2 gene was originally scored according to the 2008 guidelines on HER2 scoring in breast cancer [6]. Retrospectively, the TMA spots were evaluated again (done by Z.V.) using the modified 2013 ASCO/CAP guidelines[5].

**SISH** *HER2* signals were scored as follows: the number of signal copies and the ratios (*HER2/CEP17*) were calculated, gene copies (>6) or cluster formations (small clusters ∼6 copies, larger clusters ∼12 copies) were defined as amplified. Similarly, a ratio >2.2 was set as amplified status; a ratio <1.8 was negative, and a ratio of 1.8–2.2 was referred to as unequivocal (according the 2008 guidelines) and using ratio >2 and > or equal to 6 *HER2* gene copies (in >10% of the tumor cells) for a positive status (according to 2013 guidelines).

**Figure 1:**
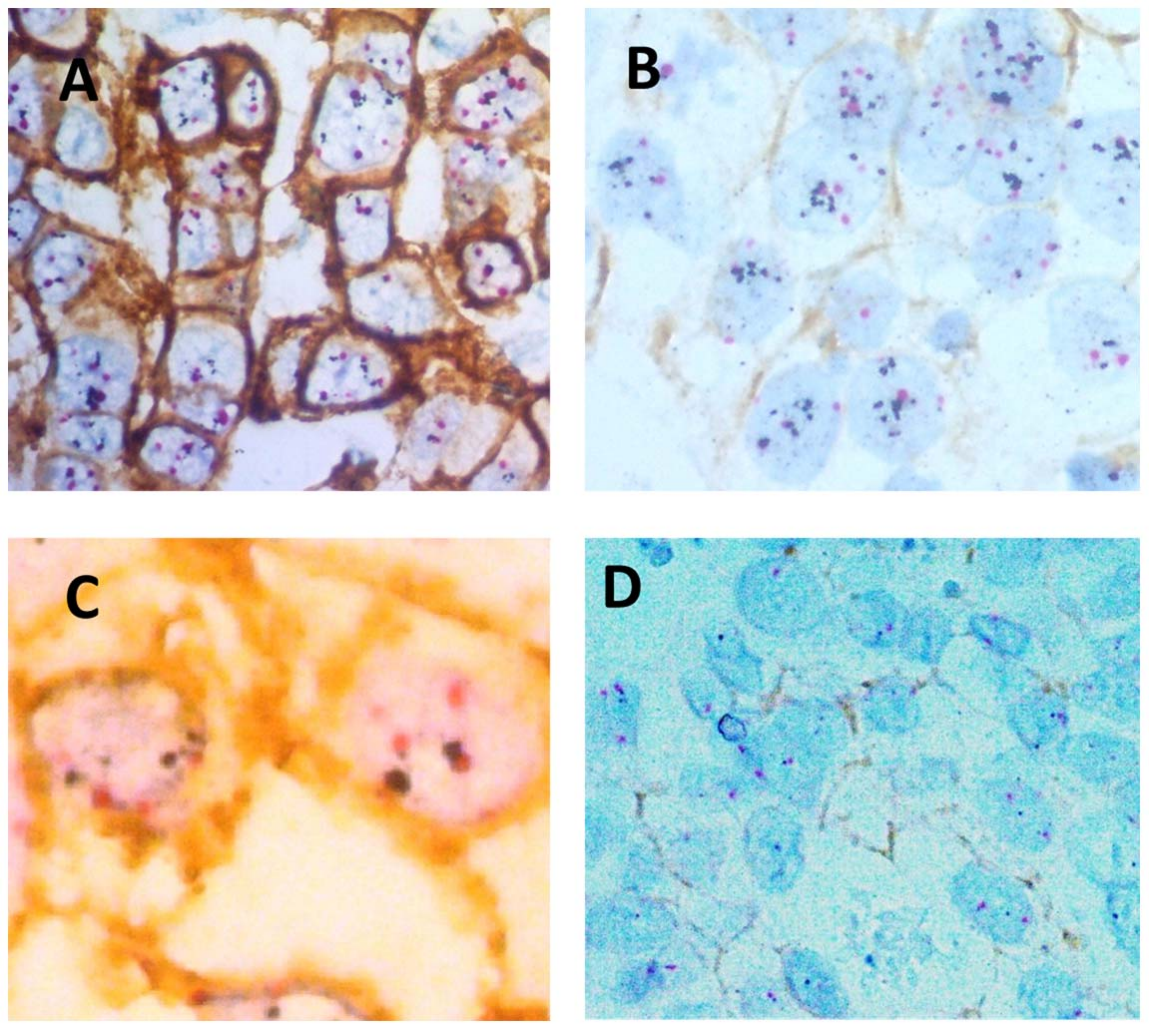
Breast cancer tumor samples with different HER2 results by double HER2 IHC/SISH. Red spots correspond to the centromere 17, black spots to the *HER2* gene. A) Sample with IHC score 3+ with simultaneous amplification of the HER2 gene by SISH. B) Another sample with IHC score 0/1+ with simultaneous amplification of the HER2 gene by SISH. C) Illustration of a case with IHC score 3+ without simultaneous amplification of the HER2 gene by SISH. D) Another case with IHC score 1+ without simultaneous amplification of the HER2 gene by SISH.

**HER2 protein expression** was scored as follows: 0 (no staining), 1+ (weak and incomplete membrane staining), 2+ (strong, complete membrane staining in less than 30% of the invasive tumor cells or weak/moderate heterogeneous complete staining in more than 10% of the invasive tumor cells) or 3+ (strong complete homogenous membrane staining in more than 30% of the invasive tumor cells). According to the 2013 ASCO/CAP guidelines, the required percentage (10%) for score 3+ and 2+ were used for the re-evaluation of the results based on the 2008 guidelines (**Fig. 1**).

### Statistical analysis

SPSS 15.0 software was used for statistical comparisons (SPSS, Inc., Chicago, IL, USA). Categorical data were analyzed using Chi- Square test. Spearman log rank, Pearson, Breslow and Tarone Ware correlation were used to correlate HER2 protein expression scores and HER2 gene copy number with and clinic-pathological parameters (stage and grade, hormone receptor status) and overall survival. Non-parametrical tests as Kendall Tau and Spearman rho were used to test correlation between overall survival and HER2 scores with IHC and SISH.

## Results

### Concordance of IHC and SISH

There were 589 interpretable spots containing sufficient amount of invasive carcinomas on the TMAs. Sixty of 589 cases were score 3+ by IHC (10%). Fifty-seven of 60 IHC score 3+ cases showed also amplification by SISH (95%). 519 of 589 cases were negative (score 0/1+) by IHC (88%). 3 of 519 IHC negative breast cancers (score 0/1+) showed amplification by SISH (0.6%). Ten of 589 samples were score 2+ by immunohistochemistry (5%), 6 of these cases displayed amplification by SISH (60%). One of the IHC score 3+ cases showed polysomy of both CEP 17 and HER2 gene (<1%) (**Table 2**).

**Table 2:** *HER2* status results by double immunohistochemistry (IHC) and silver enhanced in situ hybridization (SISH) on the tissue micros arrays (n = 589 interpretable spots).

	SISH			Total
IHC	Non amplified	amplified	polysomy	
Score 0 and 1+	516	3	0	519
Score 2+	4	6	0	10
Score 3+	2	57	1	60
Total	522	66	1	589

### Correlation of IHC and SISH scores with overall survival

**Figure 2:**
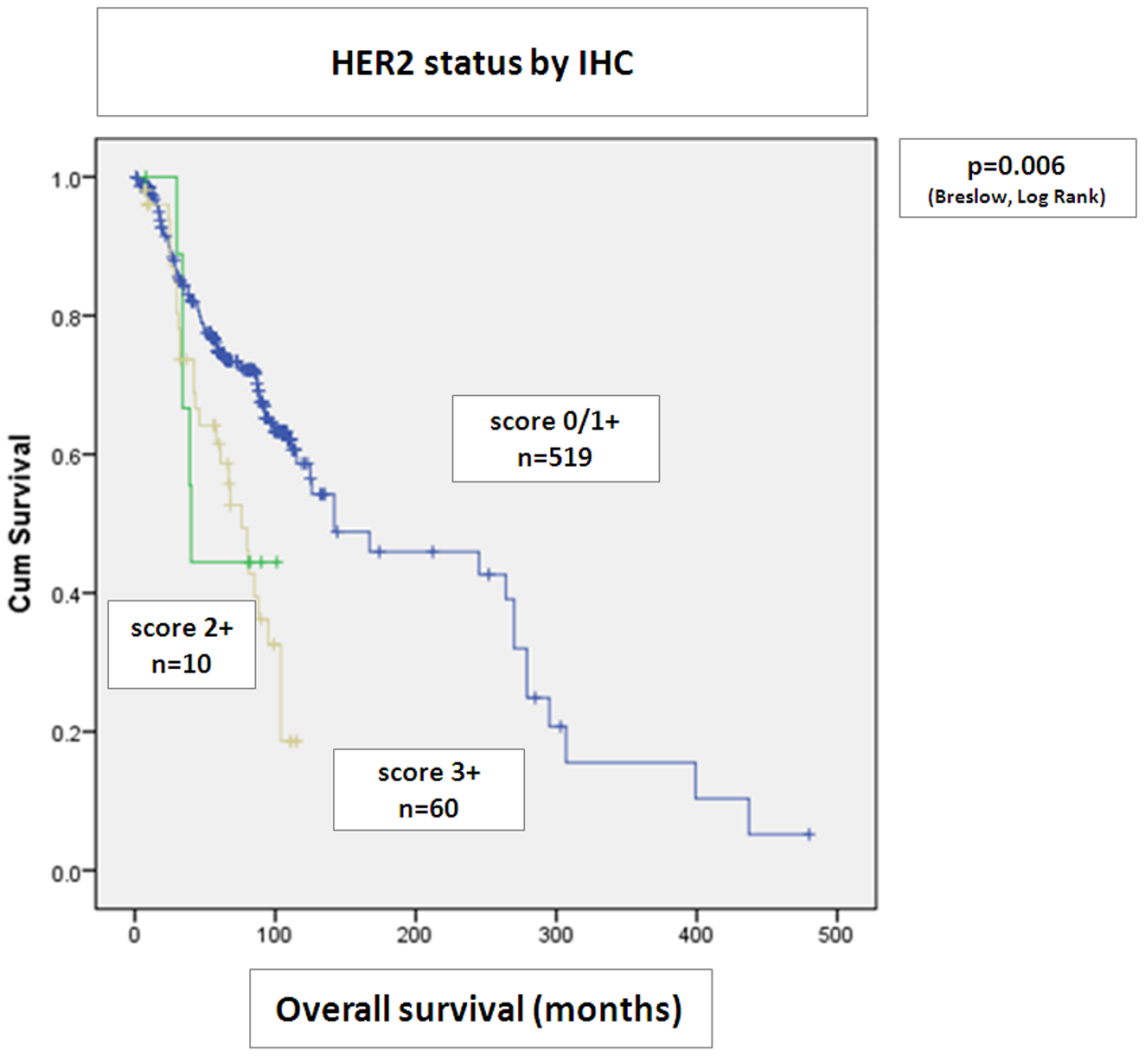
HER2 status by IHC alone. Statistical analysis with Kaplan Meier curves. IHC 0/1+, 2+ and 3+ scores significantly stratify patients in overall survival. Patients with IHC score 2+ and 3+ have significantly shortened overall survival.

**Figure 3:**
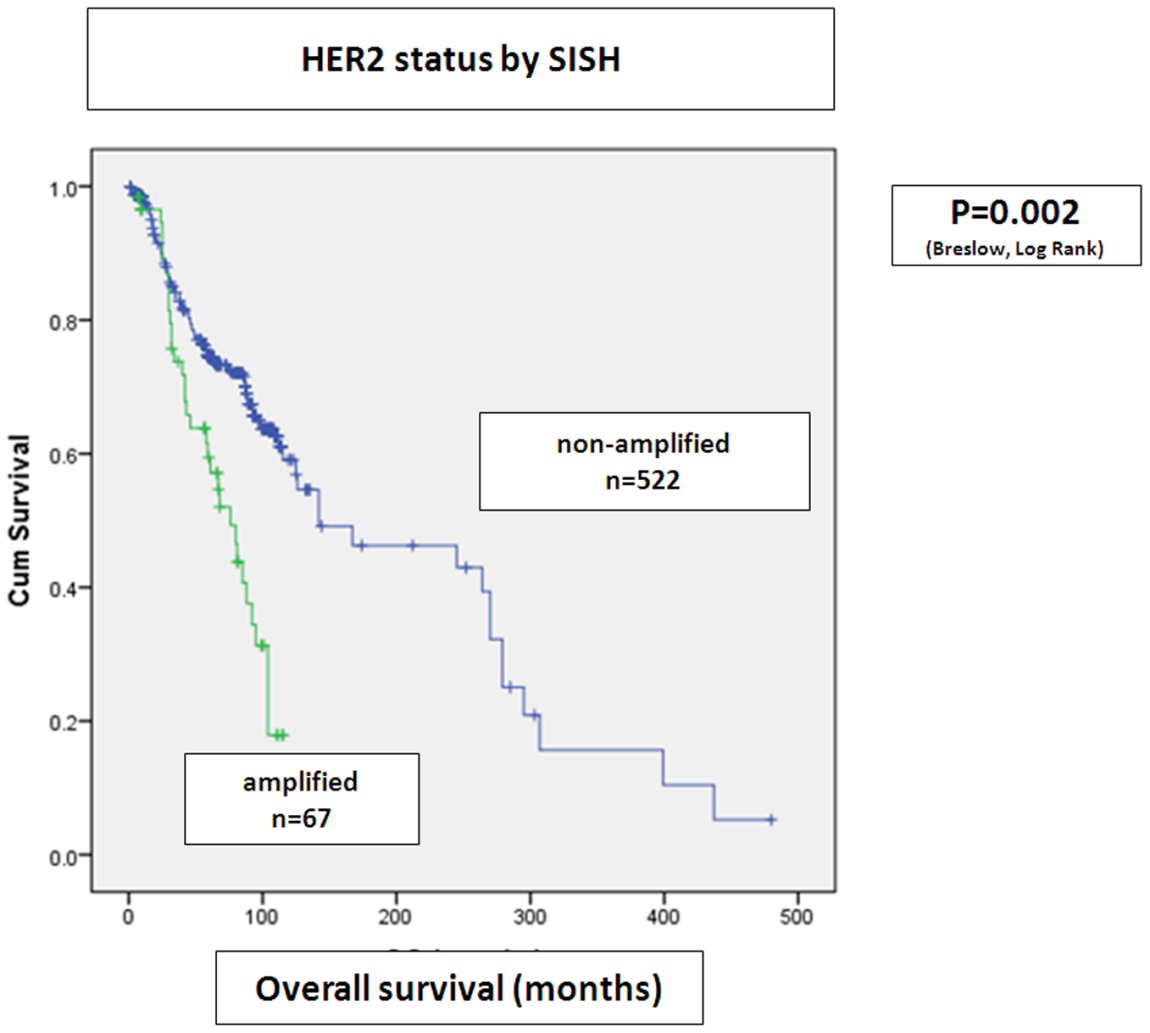
HER2 status by SISH. Statistical analysis with Kaplan Meier curves. Positive *HER2* amplification status is associated with significantly shortened overall survival.

There was a significantly shorter overall survival among patients whose tumors were either amplified by SISH (p = 0.002) or were IHC score 2+ or 3+ (p = 0.006), when these two parameters were analyzed separately (**Fig 2** **. and** **Fig 3**).

**Figure 4:**
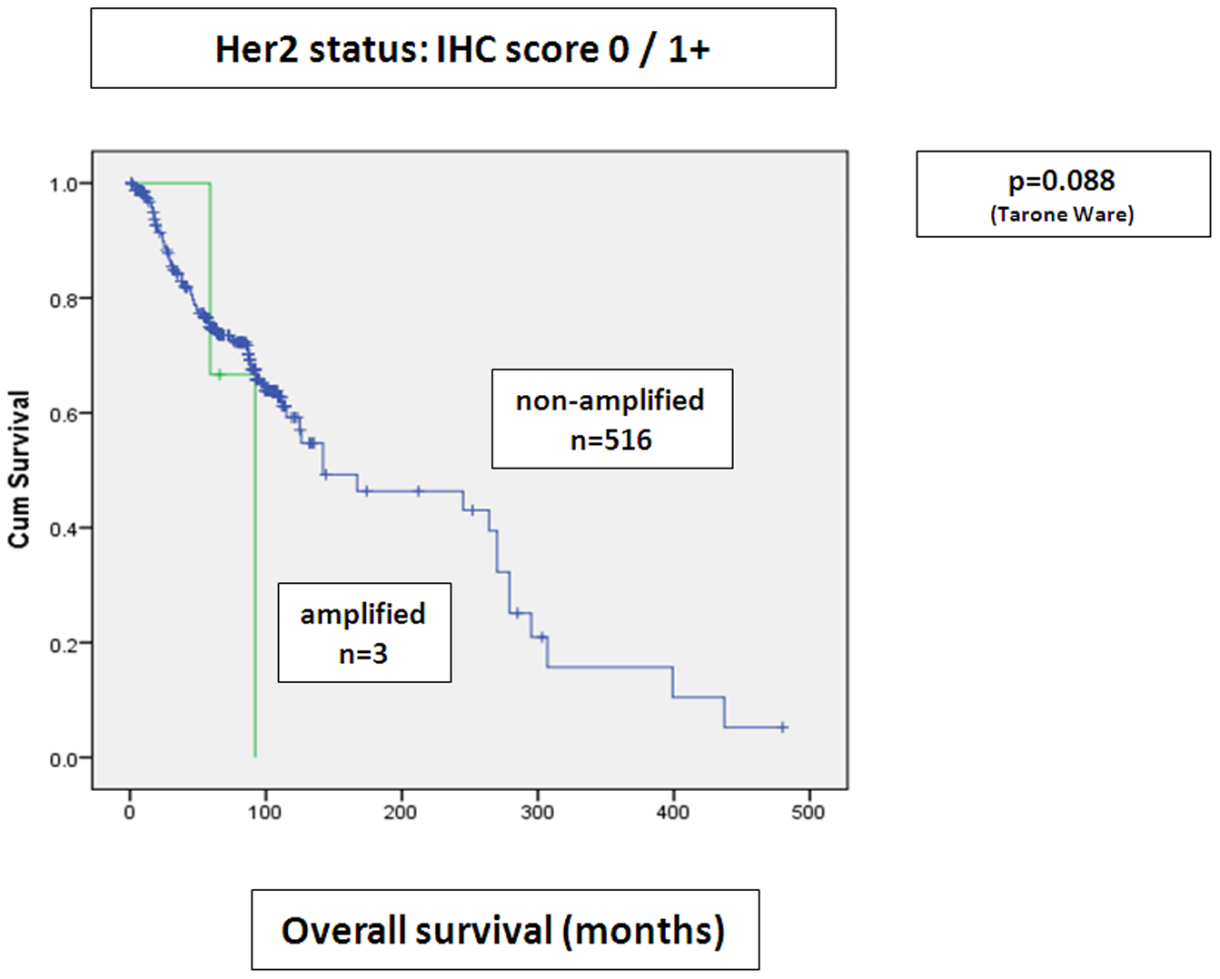
Stratification of IHC score 0/1+ tumors and amplification status by SISH to overall survival. There is a tendency to shorter overall survival in HER2 amplified IHC score 0/1+ cases (Kaplan Meier curves).

**Figure 5:**
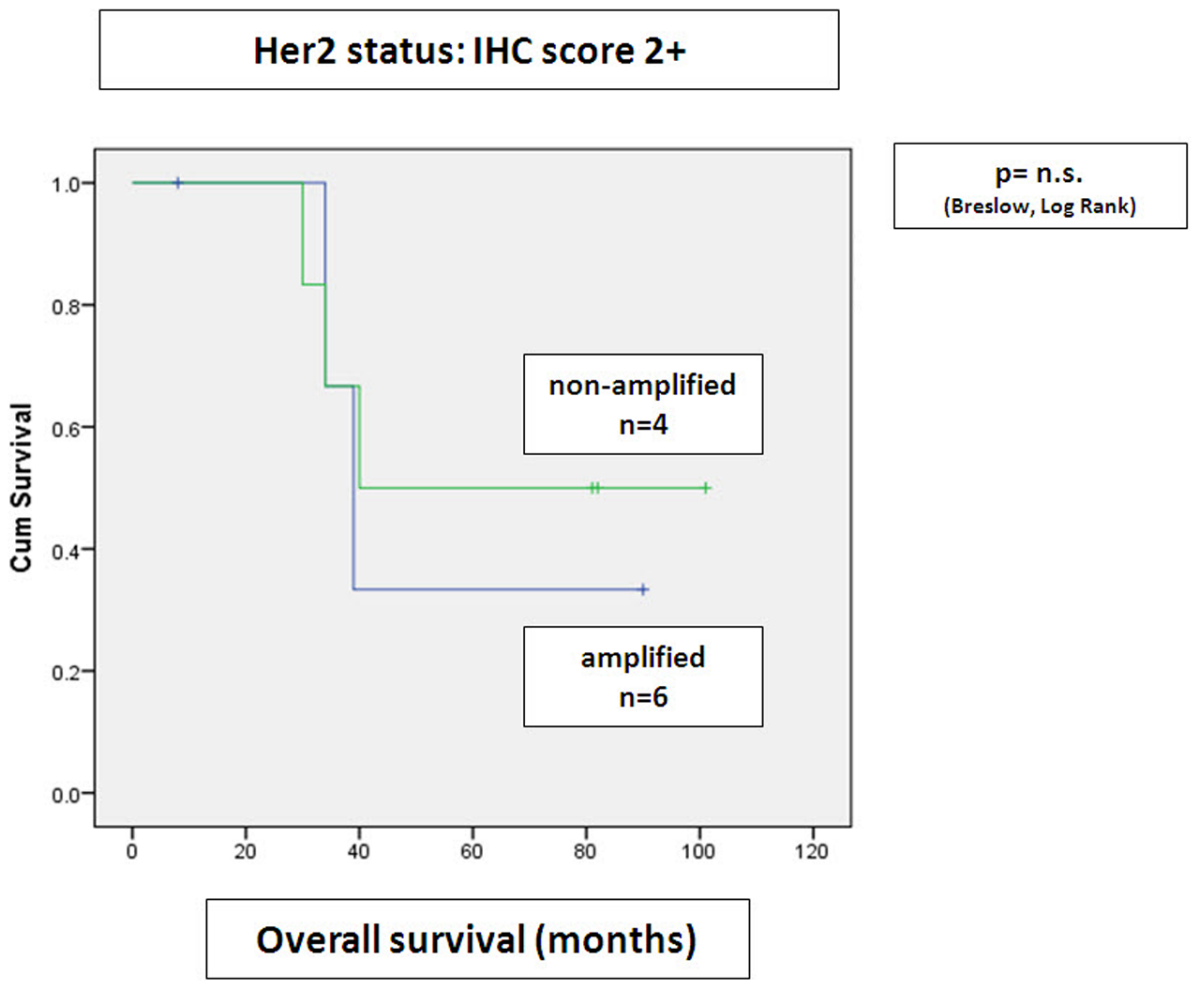
Correlation of IHC score 2+ scores and amplification status by SISH to overall survival. Amplification status in patients with IHC score 2+ had no impact on overall survival (Kaplan Meier curves).

**Figure 6:**
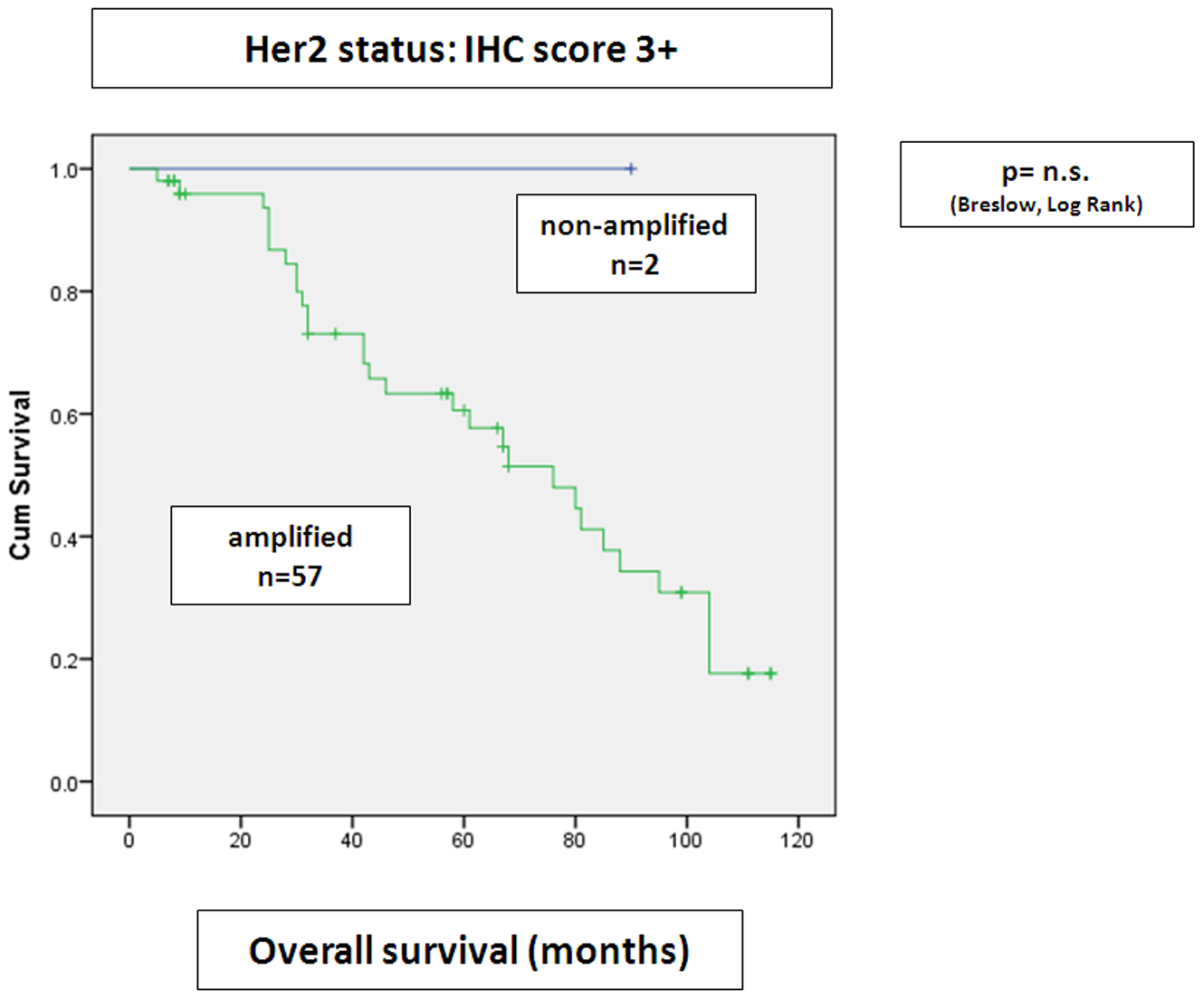
Correlation of HER2 amplification status by SISH in IHC score 3+ tumors with overall survival. Overall survival of IHC score 3+ tumors was independent from amplification status by SISH in this cohort (Kaplan Meier curves).

**Figure 7:**
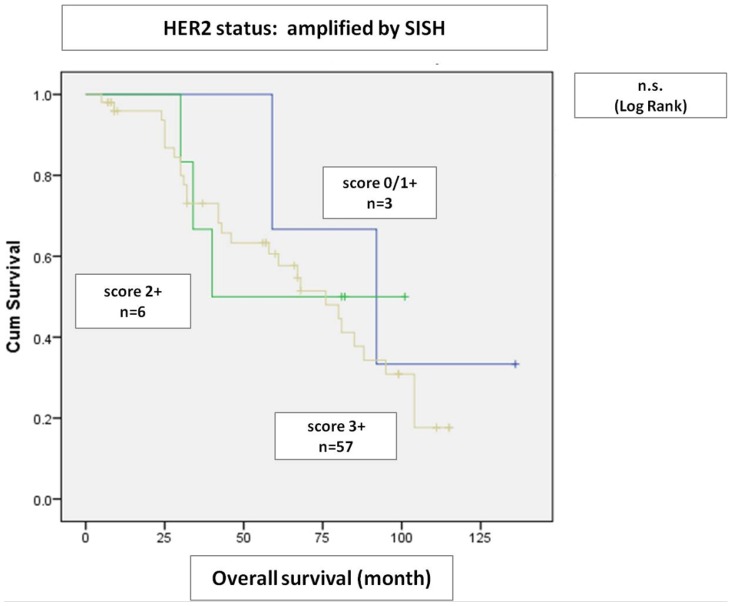
Overall survival of *HER2* amplified tumors by SISH. HER2 SISH amplified cases had a similar overall survival irrespectively from HER2 IHC scores.

When combining IHC and SISH scores, there was as tendency to shortened overall survival in patients with IHC score 1+ with simultaneous gene amplification, when compared to IHC score 0/1+ tumors without gene amplification (p = 0.088, nearly reaching statistical significance), (**Fig. 4**). No significant difference in 5 year overall survival of IHC score 3+/2+ tumors without gene amplification was seen in this cohort, when results were compared to IHC score 3+/2+ with concomitant gene amplification (**Fig. 5** **and** **Fig. 6**). Amplified cases with IHC score 1+ had a similar overall survival as IHC score 3+/2+ cases with simultaneous gene amplification by SISH (**Fig. 7**).

### Correlation of IHC HER2 and SISH *HER2* scores with clinico-pathological parameters

IHC HER2 scores significantly correlated with hormone receptor status (p = 0.001) and histological grading (p<0.001) and lacked correlation to pathological tumor stage, nodal status or to the presence of distant metastases.

SISH *HER2* scores also showed a significant correlation to hormone receptor status (p = 0.002) and to histological grading (p<0.001), but not to tumor stadium, nodal status or to distant metastases.

### Concordance of double IHC/SISH *HER2* testing to original HER2 status

Original HER2 status was available in 310 primary tumors performed by IHC and FISH technology. There were 6 of 310 (1.9%) discrepant cases with IHC and 7 of 310 (2.2%) discrepant cases with FISH as compared to double IHC/SISH HER2 testing.

## Discussion

We analyzed HER2 status in a cohort of invasive breast cancers using a double IHC/SISH assay, enabling the simultaneous detection of the HER2 protein expression and the *HER2* gene copy number. Importantly, the simultaneous detection of HER2 protein and *HER2* gene copy number facilitates the identification of relevant prognostic subgroups with poor prognosis especially in the IHC score 0/1+ group with amplification and in the IHC score 3+/2+ group without gene amplification.

The novel technology of bright field protein and gene detection assay was developed and described in 2004 by Tubbs et al [12], [13]. This innovative approach for simultaneous detection of protein overexpression and gene copy number provides several advantages over established FISH and IHC based technologies [12], [13]. FISH is a precise yet robust analysis, which needs thorough technical expertise both in localizing and interpreting the signals [12], [13]. The bright field in situ hybridization technology, originally named as EnzMet, today known as SISH (silver enhanced in situ hybridization) is an enzyme metallographic probe labeling, resulting in silver coloration of the hybridized gene sequence. This provides a higher sensitivity and higher resolution of the amplified and non-amplified gene regions, which can be visualized and analyzed on the light microscope [12], [13]. The advantage of a permanent result and the possibility of bright-field microscopy make SISH an attractive alternative to FISH. We have recently demonstrated a high concordance of SISH and FISH results in the detection of EGFR copy number alterations in lung cancer samples and we have provided data that SISH is an accurate method for the evaluation of the Her2 gene amplification status in cytologic breast cancer specimens, particularly in metastatic breast cancer lesions [16] [17].

In our TMA-based study, we demonstrate an excellent correlation between IHC score 3+ and gene amplification by SISH using a dual probe for IHC and SISH. Concordance between IHC and ISH technologies in HER2 status assessment in breast cancer has been the subject of several previous studies [3], [6], [18], [19]. These studies confirmed a realistic concordance between IHC and SISH technology, especially in settings where ISH is applied as reflex testing in IHC score 2+ cases. In order to keep necessary acceptable quality standards, the ASCO/CAP guidelines have set a minimum of 95% concordance between IHC and ISH technologies, when both methods are used [6], [20]. The need for standardized methodologies in HER2 testing, including not only internal quality assurance procedures but also test validation, sufficient fixation time, proper training of involved technical and medical staff persons, have been added to several guidelines [7], [18], [20]–[25]. Although fixation time might be different in samples in a TMA cohort, this was obviously not significantly affected by analytic laboratory and interpretational issues.

Here, we demonstrate that the novel bright-field ISH assay enables improved diagnosis in borderline breast cancer cases. Simultaneous assessment of HER2 status by IHC and SISH shows a significant correlation to overall survival. The use of different ISH assay for the assessment of the HER2 status was assessed in several previous works [2], [18], [26]–[31]. These works demonstrate that FISH based HER2 assays and the bright field ISH technologies (as silver or chromogenic enhanced technologies) provide highly concordant information on HER2 status. These alternative ISH tests as CISH or SISH have shown a good to excellent correlation between the tests themselves, and IHC score 3+ and ISH amplified cases, varying from 89% to 98% [2], [18], [26]–[31]. According to our knowledge, only one of these studies examined simultaneously the HER2 protein expression by performing IHC prior to SISH on the same slide in a cohort of gastric and breast cancer cases [31].

Our study confirms existing data that IHC score 2+/3+ tumors as well as tumors with *HER2* gene amplification represent prognostically poor subgroups with shortened overall survival [7], [20], but our results are of importance for recommendations using either FISH or IHC alone. Given the low rate of IHC score 0/1+ cases, many breast cancer centers recommend FISH as a primary and only test assay for HER2 analysis (REF). We have recently reported that HER2 positivity rate remained stable by FISH-technology, but showed a significant variation by IHC in a single institution over 12 years [32]. This can be considered as a further argument for a FISH alone test approach. However, our results obtained with the novel double immunohistochemistry (IHC) and silver labeled in situ hybridization (SISH) assay demonstrated that patients with IHC score 3+/2+ with and without amplification have the same survival. The FISH only test approach would have missed patients with IHC score 3+/2+ without amplification with the consequence that these patients would have not received Trastuzumab therapy.

The underlying mechanism of protein overexpression in breast carcinomas is preferential amplification or polysomy of the *HER2* gene. This amplification can vary in size, especially in IHC scores 3+ cases [7], [20]. It is well documented that IHC score 3+ cases may lack gene amplification. This phenomenon is reported in up to 20% of tested cases and can be due to pre-analytical and interpretational factors including false-negative polysomy [3]–[5], [7], [19], [33]. In some instances, protein overexpression occurs as the result of true aneuploidy with numerical changes of the whole chromosome 17, but this phenomenon is preferentially seen in HER2 IHC scores 1+/2+ tumors. Both mechanisms can occur also concomitantly within the same tumor, potentially yielding further interpretational issues. The double immunohistochemistry and silver labeled in situ technology is able to separate high level amplifications and/or polysomy of chromosome 17 as relevant mechanisms for HER2 overexpression in tumor cells. In our cohort, we found a very low incidence of real polysomy, only one of 589 cases showed polysomy with SISH and displayed IHC score 3+, This observation is indeed consistent with current recommendations as to precise handling and interpretation of polysomy in HER2 status [1], [4], [5].

As to the real frequency of IHC score/1+ cases having simultaneous amplification, the literature is inconsistent, mostly due to the fact, that IHC score 0/1+ cases do not need to undergo obligatory reflex testing with an ISH technology [6], [19], [23]. Nevertheless, this phenomenon can be seen in up to 10% of breast cancer cases [23], [26]–[28], [30], [34]. It is to assume, that pre-analytical factors as poor fixation or laboratory procedure errors contribute to a false-negative or weak signal in IHC and can explain why gene amplification do occur in IHC score 0/1+ cases [7], [23]. The prognostic/predictive role of IHC score 0/1+ with amplification has not been studied extensively [8], [10], [11]. The recently closed HERA trial could show that neither IHC staining intensity nor the ratio of FISH testing had significant impact on response to Trastuzumab therapy in HER2 positive disease. Results of conflicting outcome data raise the role of possible pre-analytical inconsistencies in HER2 status assessment as well [8], [11]. On the other hand, the N9831 trial, provided lack of evidence on benefit on Trastuzumab and/or on survival benefit in HER2 negative (IHC and/or FISH) breast cancer [10]. An earlier paper of Mass et al. found a benefit for Trastuzumab therapy only in FISH positive tumors [9]. In our cohort, we could identify three patients with IHC score 0/1+ with simultaneous gene amplification by SISH. Even though this is a small number, these three patients had a similar survival curve as patients with IHC score 3+ and amplification. Interestingly, IHC score 2+ cases in our cohort displayed a shortened survival independently from the presence of gene amplification. The relatively low IHC score 2+ rate with a high concomitant amplification rate in our cohort is most likely explainable due to the unbalanced study population in the tissue micro-arrays.

Several problematic topics in routine HER2 testing prompted the revision of the 2007 ASCO/CAP guidelines on HER2 diagnostic guidelines. Diversities in the interpretation of chromosome 17 aneuosomy and of HER2/CEP17 ratios may lead to false negative and false positive HER2 status, having an immediate impact on therapy options for the given patient. Tumor heterogeneity, biological characteristics and pre-analytic issues have been also shown to influence accuracy in HER2 status assessment [5] [32]. As addressed also in our study, the identification of prognostically relevant subgroups in breast cancer is of utmost importance, which requires established precise technologies and interpretation.

Our cohort contained also samples from metastases but not from the primary lesions. In metastatic lesions, there was only one case with discordant HER2 protein and *HER2* gene status. Concordance in HER2 status between primary tumors and metastastic sites was the subject of several previous studies, confirming a high consistency in cases with only one metastastic site [7], [20]. At the same time, changes in HER2 status were reported in metastatic breast cancer patients with multiple metastatic sites and has also been observed in breast cancer samples after adjuvant or neoadjuvant chemotherapy [7], [20].

## Conclusions

We provide data that *HER2* gene amplification independently from HER2 protein expression in IHC score 0/1+ cases correlates with tendency to poorer overall survival. Importantly, our data suggest that the double immunohistochemistry and silver labeled in situ hybridization assay identifies a prognostically relevant IHC 2+/3+ breast subgroup without HER2 amplification, which cannot be detected using a FISH alone approach.
